# PolyprOnline: polyproline helix II and secondary structure assignment database

**DOI:** 10.1093/database/bau102

**Published:** 2014-11-06

**Authors:** Romain Chebrek, Sylvain Leonard, Alexandre G. de Brevern, Jean-Christophe Gelly

**Affiliations:** ^1^Inserm U1134, Paris, France, ^2^Université Paris Diderot, Sorbonne Paris Cité, UMR_S 1134, Paris, France, ^3^Institut National de la Transfusion Sanguine, Paris, France and ^4^Laboratory of Excellence GR-Ex, Paris, France

## Abstract

The polyproline helix type II (PPII) is a regular protein secondary structure with remarkable features. Many studies have highlighted different crucial biological roles supported by this local conformation, e.g. in the interactions between biological macromolecules. Although PPII is less frequently present than regular secondary structures such as canonical alpha helices and beta strands, it corresponds to 3–10% of residues. Up to now, PPII is not assigned by most popular assignment tools, and therefore, remains insufficiently studied. PolyprOnline database is, therefore, dedicated to PPII structure assignment and analysis to facilitate the study of PPII structure and functional roles. This database is freely accessible from www.dsimb.inserm.fr/dsimb_tools/polyproline.

## Introduction

Fifty percent of local protein conformations are constituted of the two regular secondary structure, i.e. α helices and β sheets, while the remaining protein structure is essentially constituted of turns that can overlap the two previous local conformation, and coil (1). Regular secondary structures are fundamental descriptor for the analysis and the understanding of the structure and function of proteins at a molecular level. As such, they are automatically used to visualize the protein 3D structures with popular software like PyMOL (2), VMD (3) or Chimera (4). Thus, the secondary structures assignment is an essential step for studying protein architecture, folding and for the prediction of 3D protein structure. Besides α helices and β sheets, a number of other regular secondary structures are often ignored, despite their importance in biological processes (5). Among other regular secondary structures the polyproline II helix (PPII) is of significant interest. PPII conformation was primarily identified in the 1950s in collagen helix by Pauling and Corey (6), and in structures containing many repeating proline amino acids (7). It was not until the end of the 1990s that this conformation has been demonstrated to occur frequently in globular protein (8), with a very high conservation ratio of 80–100% in proteins families sharing 20% sequence identity or more, a ratio close to the conservation found for α helices and β strands (9). Depending on the tools used for the assignment of secondary structures, their frequency varies in the range of 3–10% of all conformations with a common core of more than 1.6% assignment shared by all tools (10). Other studies have shown similar frequency, Adzhubei and co-workers in a recent review (11) estimated about 2% of residues in Protein Databank to be in PPII-helices of length 3 and more residues. For historical reasons, this conformation had been called ‘polyproline helix’, although most PPIIs comprised non-proline residues and some even contain no proline at all (8).

In term of local structure conformation, Polyproline II is a left-handed helical conformation with average dihedral angle values of Φ = −75° and Ψ = +145°. Unlike classical regular secondary structures, PPIIs are not usually associated with conventional stabilizing internal hydrogen bonds due to this extremely extended conformation. PPII is a far more extended helix than classical α-helix (5.4 Å/turn, 3.6 residues per turn) and has a helical pitch of 9.3 Å/turn and 3 residues per turn. Thanks to this over extended conformation and high solvent exposure, residues in PPII may lead to potential interactions with other molecular partners. Thus, it was suggested that they might have an important functional role, particularly in protein–protein or protein–nucleic acid interactions and recognition (12, 13). Regrettably, PPIIs are still insufficiently studied. In fact, PPII assignment is not done with the most common method of secondary structure assignment such as Dictionary of Protein Secondary Structure (DSSP; 14) and STRIDE (15), and therefore, newly solved protein structures are not assigned with PPII in Protein DataBank (16). Here we introduce a new assignment method and a dedicated webserver for PPII.

## Aim and overview of database

The PolyprOnline database (http://www.dsimb.inserm.fr/dsimb_tools/polyproline) contains secondary structure assignments on a large subset of the Protein Databank. It also allows to dynamically handle any new user submitted structures. Unlike other databases established for protein secondary structure analysis, PolyprOnline particularly focalize on PPII, an assignment that is rarely documented in experimentally solved structures as well as in services and tools dedicated to the analysis of protein structures. For instance, 2struc (http://2struc.cryst.bbk.ac.uk/about/) assigns protein in three secondary states using six different algorithms (17), but none of them address PPII assignment. More general tools such as PDBsum (18) give assignment by one method, PROMOTIF (19) in this case, with no details about PPII. As previously mentioned, this assignment is especially important since this conformation is the third most abundant regular secondary structure just behind α-helix and β-strand, and it is also involved in various function related to molecular interactions such as protein–protein and protein–nucleic-acid binding. However assignment using different tools show discrepancy thus our database provide assignments with the four main methods developed so far (10).

## Results and features

**Figure 1:**
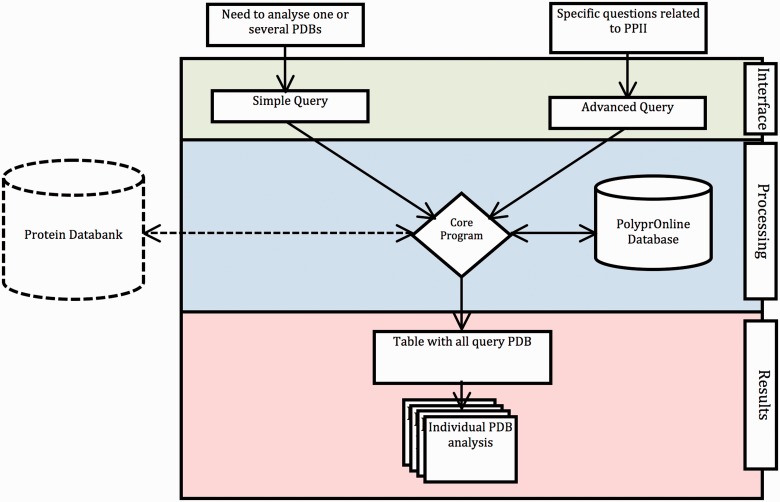
Data flow in PolyprOnline system. Access to the system can be done in two ways: through ‘Simple query’ for the analysis of one or more protein structures from their PDB code and through ‘Advanced query’ for performing more complex queries using different criteria such as resolution (Å), protein length, minimal and maximal number or percentage of residues in PPII conformation assigned by a particular tool. The last type of advanced query allows local structure search on specific positions using secondary structure patterns. It is also possible to dynamically upload and process a PDB file if it is absent of the database. The query is then processed to be interpreted by our Database Management System. In the case where a PDB structure is not found in the database, a PDB file can be downloaded from the Protein Databank website and dynamically processed by the system. PolyprOnline webserver offers the following outputs to display results: Summary of all protein identified by PDB code, title, size, resolution and PPII content, printed in a sortable table according to the values in different columns (Figure 2). From this table, individual protein data analysis can be accessed individually (Figure 3).

The data flow and processing step performed by the system are summarized in Figure 1.

### Interface

Through the main interface, two types of search are possible. Both searches are detailed in text of Figure 1: simple search (analysis of one or more protein structure) and advanced search based on specific criteria to perform more complex queries. One of the most interesting features is the ability to perform secondary structure pattern query. This search is useful to look for a fragment of specified conformation contained in protein structures using a simple regular expression pattern. Pattern search uses the classical rules for regular expressions. It is possible to use conformation code letters (e.g*.* HHHH-PPEEE), and introduce wildcard (e.g*.* HHH**PP*-). It is also possible to specify the minimal and the maximal conformation length (e.g*.* PPPX{1,8}PP).

### Outputs

The PolyprOnline webserver offers the following outputs:

#### A table of sortable results

**Figure 2:**
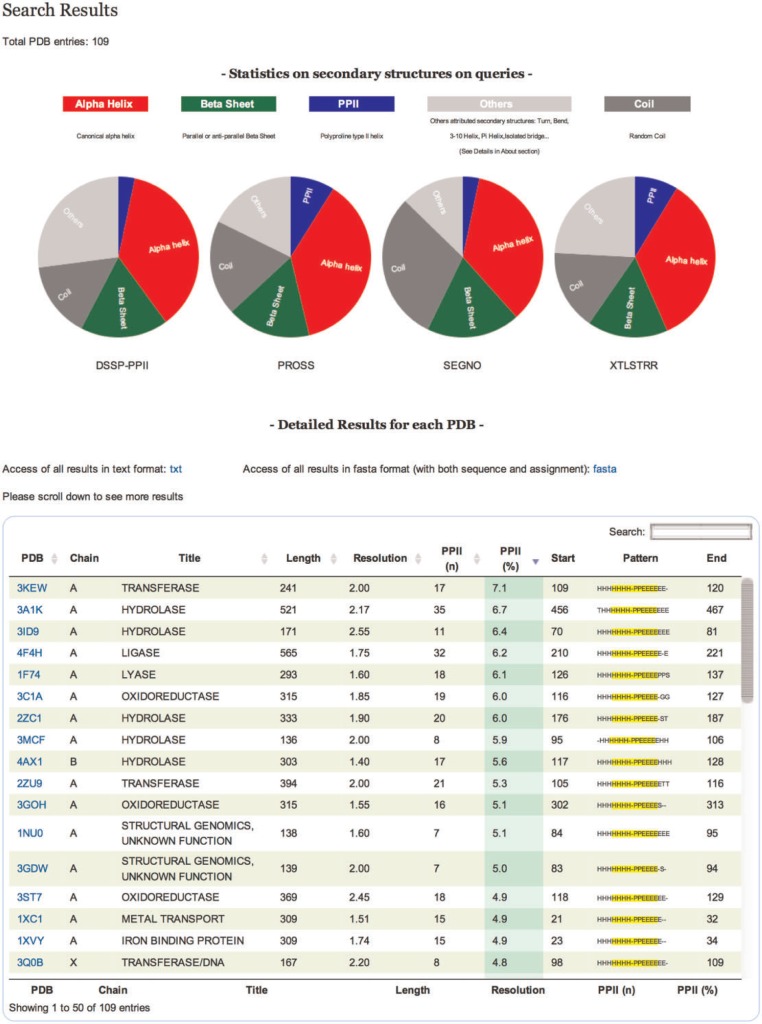
Results. On the top of the table a pie chart displaying statistics of secondary structure content for each tool of all entries is dynamically generated. The table gives information on each selected protein chain. Each line corresponds to a PDB chain and each column to attribute values describing every entry. Alphabetical (PDB, Title) or numerical (length, resolution, PPII number and percentage) ordering and re-ordering of entries in ascending or descending order is possible. Another possibility is to do a free text search through a specific field. Each detailed analysis can be accessed from this table.

Results are displayed in a table that can be sorted accordingly to the values in different columns (Figure 2). Results in the table can also be directly downloaded in text format. All proteins in the table are identifiable by PDB code, title, size, resolution and PPII content. You can also download the assignment of each protein in classical fasta format.

#### Individual protein data and analysis

**Figure 3:**
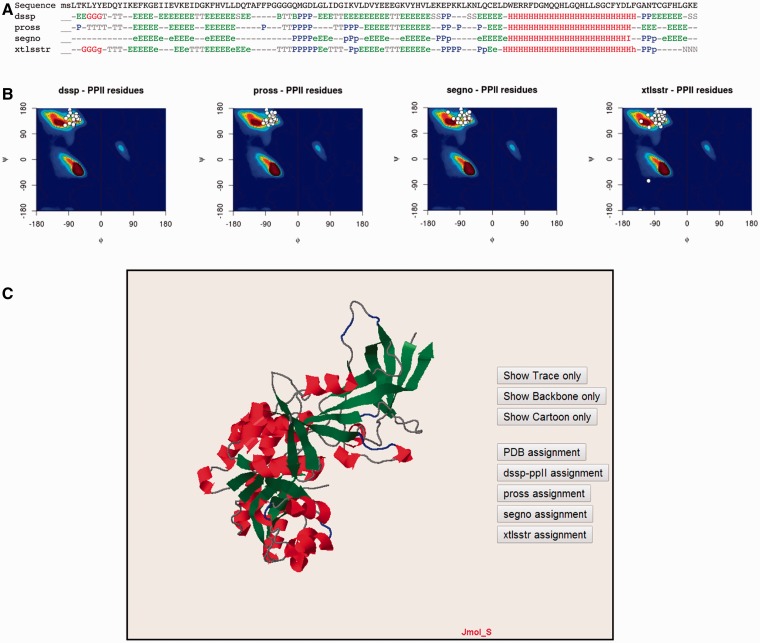
Detailed analysis of a protein structure (3KWEA; 25). (**A**) Sequence and analysis of secondary structures using four different protein secondary structure assignment methods are printed on a 1D alignment. One letter code is used to represent a specific conformation. Letters are coloured accordingly to more general class of secondary structure (i.e. helix residue in red, strand in green, PII helix in blue and non-regular secondary structure in grey). (**B**) Ramachandran plots give the distribution of φ and ψ torsion angles of PPII amino acids for each method. The most frequent areas for α-helix and β-sheet are shown in the background of the plot (represented by a colour scale). Statistics about areas were derived from our previous study. Residues assigned as PPIIs are represented as white points. (**C**) Full 3D structure visualization and animation using a JMol applet of different assignment can be dynamically displayed (Cα trace only, cartoon). Local conformations are coloured with the same colour scheme as used for the 1D alignment in (A; i.e. helix residue in red, strand in green, PII helix in blue and non-regular secondary structure in grey).

The PolyprOnline web server provides access to different assignment methods and allows visualization of both regular secondary structure and PPII helix (Figure 3). We have recently underlined the discrepancies between the three different secondary structure methods able to assign PPIIs, and proposed a novel PPII assignment using the de facto standard DSSP assignment method (10, 14). To better visualize the secondary structure and PPII assignments given by PROSS (21), SEGNO (22), XTLSSTR (23) and our DSSP-PPII (10, 14), they are all displayed at the bottom of sequence One letter code is used to represent specific conformation. Letters are coloured accordingly to more general class of secondary structure (e.g. helix residue in red, strand in green, PPII helix in blue non-regular secondary structure in grey, coil being in dark grey colour) for a fast visualization of overall local structures. All data from protein structure analysed can be downloaded.

Ramachandran plots give the distribution of φ and ψ torsion angles for each assignment method. The most frequent areas for α-helix and β-sheet are shown in the background of the plot (represented by a colour scale). Statistics about areas were derived from our previous study (10). Residues assigned as PPIIs are represented as white points. The image is mouse sensitive and gives additional information on residue number, nature and φ and ψ angle values of assigned as PPII. Indeed assignments provided by the various tools can be quite different between them. Ramachandran plot lets to visually inspect φ and ψ angle PPII value distributions and help the user to apprehend the relevance of each assignment.

Visualization and manipulation of three dimensional protein structures is allowed thanks to a JMol applet (24). It displays the assignment of secondary structures by all of the four methods and details about positions of secondary structures with a particular emphasis on PPII. This visualization can also be useful to observe difference between assignments directly in protein structure.

## Methods

### Protein structures dataset

A subset of the experimental protein structures extracted from the PDB was selected based on the resolution methods (RX), quality of structures (resolution lower than 3.0 Å and R-factor lower than 1.0) limited redundancy (proteins share no more than 90% of identity between each others) using webserver PISCES (20). The full list of selected structures comprised 24 761 protein chains and is available on database. The list is regularly updated.

### Assignment of PPII and other secondary structures

Currently, there is a limited number of tools for assigning PPII number. The tools available today are XTLSSTR, PROSS (version September 2004) and SEGNO (version 3.1). We have added our PPII DSSP-based program DSSP (CMBI version 2000) developed in our laboratory to this list (10). As we have previously explained, the use of multiple tools is necessary because it has been shown that PPII assignments using several methods yielded different results (10).

Secondary structures assigned by PROSS (21) are as follow: α helix (H), β turn (T), β strand (E), PPII (P), and coil (C). Assignments are based exclusively on Φ and Ψ dihedral angles.

The algorithm XTLSSTR (23) uses two angles and three distances to assign secondary structure from coordinates of PDB files. It assign secondary structures: α helix (H and h), 3_10_ helix (G and g), hydrogen bonded β turn (T), non-hydrogen-bonded β turn (N), Extended β strand (E and e) and PPII (P and p)

SEGNO (22) uses also the Φ and Ψ dihedral angles coupled with other angles to assign the secondary structures. It assign α helix (H), β-strand (E and e), isolated β-strand (B and b) 3_10_ helix (G and g), π-helix (I), coil (O, coded as ‘-’ in this database) and PPII (P and p).

DSSP-PPII is a new method for PPII assignment recently developed in our laboratory (10). It is based on the most popular secondary assignment tools: DSSP (14). DSSP assignment is based on the identification of precise hydrogen bond patterns corresponding to regular secondary structures. Assignment strategy of PPII is based on simple set of basic rules to have the highest agreement with PROSS, SEGNO and XTLSSTR methods. PPII are assigned solely in the coil region for at least two consecutive amino acids in coil with Φ = −75° ± *ε* and Ψ = +145° ± *ε* with *ε* = 29°. Basic assignment of secondary structure in DSSP defines eight types of secondary structures: α helix (H), extended β strand in parallel and or anti-parallel β-sheet conformation (E), isolated β-strand (B), 310 helix (G), Pi helix (I), bend (S) and coil (O, coded as ‘-’ in this database). This is the basic assignment to which helix PPII (P) has been added.

### Web interface and Database

Database management server used by our system is MySQL. The PolyprOnline web interface has been written mainly in PHP, Perl, R and Javascript programming languages.

## Conclusion and interesting case study

**Figure 4:**
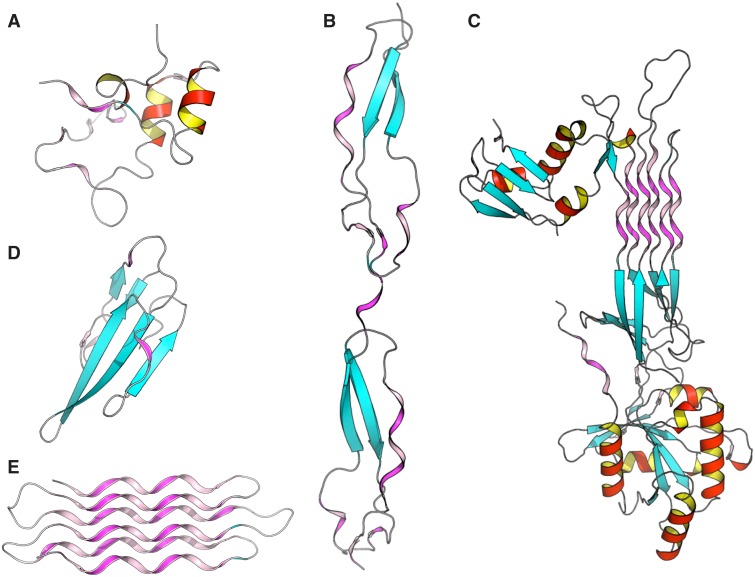
Some examples of proteins with high number of PPII conformations revealed by PolyprOnline database. (**A**) Cyclin-dependant kinase regular subunit (1CKSA; 26), (**B**) Thrombospondin (1LSLA; 27), (**C**) GTP-binding protein OBG (1UDXA; 28), (**D**) Atratoxin (1V6PA; 29) and (**E**) Snow flea anti-freeze protein (2PNEA; 30). β sheets appear in cyan while α helices are in red with an internal face in yellow. PPII are in violet and pink for internal face. Some PPII arrangements are very well organized in anti-parallel six helix bundle such in Snow anti-freeze protein (**E**) or in GTP-binding protein OBG (**C**). Others architectures are remarkable: β-β-PPII or PPII-β-β architecture found in Thrombospondin (**B**) and Atratoxin (**D**) have a similar arrangement to well known motif β-β-α or α-β-β building with an α helix instead of PPII. Cyclin-dependant kinase regular subunit (**A**) does not show any PPII specific arrangement.

To better understand structure/function and structure/architecture relationships, the advanced search interface of PolyprOnline can be used to find proteins with a high content of PPII. Thus a query launched on the basis of PPII frequency or containing long PPII helix can highlight different properties and peculiarities. It can be noted that proteins with the highest content of PPII have an over-frequency of functions related to interaction mechanisms and/or binding, which is consistent with observations in (11). For example, Figure 4 provided some examples involved in various function such as cell adhesion (B), self binding (C) or binding to cyclin-dependent kinases (A), neurotoxicity, an effect that involved blockade of acetylcholine receptors (D) and anti-freeze effect where solvent interaction is fundamental (E). With more than 72% of residues in PPII conformation, this anti-freeze protein contains the highest percentage of PPII of our database. It can also be noted, in these examples, that the organization of these PPII present characteristics of this regular conformation: rather isolated and exposed prolines for cyclin-dependant kinase regulation subunit (A), and the characteristics of other regular secondary structures: (i) similarities with α helix motifs such as PPII-beta-beta motif in Thrombospondin (B) and Atratoxin of cobra venom (D), (ii) and analogy with both alpha and beta motif such in GTP-binding protein obg (C) and snow flea anti-freeze protein (E) where PPII arrangements appear as a six anti-parallel PPII helices bundle. All theses PPII have in common a broad exposure to the solvent as it has already been highlighted in previous studies (11). Please note that these proteins are extreme cases in term of PPII content and are provided for illustrative purposes. The largest continuous PPII helix, of 13 residues long, is found in a protein Lyase (2VK8A; 31). This quick analysis highlights the utility of PolyprOnline database for PPII study.
